# Combined single-step evaluation of functional longevity of dairy cows including correlated traits

**DOI:** 10.1186/s12711-023-00839-6

**Published:** 2023-10-25

**Authors:** Laure-Hélène Maugan, Roberta Rostellato, Thierry Tribout, Sophie Mattalia, Vincent Ducrocq

**Affiliations:** 1 Université Paris-Saclay, INRAE, AgroParisTech, GABI, 78350 Jouy-en-Josas, France; 2 GenEval, 3 Rue du Petit Robinson, 78350 Jouy-en-Josas, France; 3 Idele, 78350 Jouy-en-Josas, France

## Abstract

**Background:**

For years, multiple trait genetic evaluations have been used to increase the accuracy of estimated breeding values (EBV) using information from correlated traits. In France, accurate approximations of multiple trait evaluations were implemented for traits that are described by different models by combining the results of univariate best linear unbiased prediction (BLUP) evaluations. Functional longevity (FL) is the trait that has most benefited from this approach. Currently, with many single-step (SS) evaluations, only univariate FL evaluations can be run. The aim of this study was to implement a “combined” SS (CSS) evaluation that extends the “combined” BLUP evaluation to obtain more accurate genomic (G) EBV for FL when information from five correlated traits (somatic cell score, clinical mastitis, conception rate for heifers and cows, and udder depth) is added.

**Results:**

GEBV obtained from univariate SS (USS) evaluations and from a CSS evaluation were compared. The correlations between these GEBV showed the benefits of including information from correlated traits. Indeed, a CSS evaluation run without any performances on FL showed that the indirect information from correlated traits to evaluate FL was substantial. USS and CSS evaluations that mimic SS evaluations with data available in 2016 were compared. For each evaluation separately, the GEBV were sorted and then split into 10 consecutive groups (deciles). Survival curves were calculated for each group, based on the observed productive life of these cows as known in 2021. Regardless of their genotyping status, the worst group of heifers based on their GEBV in 2016 was well identified in the CSS evaluation and they had a substantially shorter herd life, while those in the best heifer group had a longer herd life. The gaps between groups were more important for the genotyped than the ungenotyped heifers, which indicates better prediction of future survival.

**Conclusions:**

A CSS evaluation is an efficient tool to improve FL. It allows a proper combination of information on functional traits that influence culling. In contrast, because of the strong selection intensity on young bulls for functional traits, the benefit of such a “combined” evaluation of functional traits is more modest for these males.

**Supplementary Information:**

The online version contains supplementary material available at 10.1186/s12711-023-00839-6.

## Background

Genetic evaluations using pedigree and phenotypic information have been used for decades to estimate the breeding values of animals. However, low-heritability traits are difficult to evaluate accurately. Moreover, in some cases, phenotypes that are recorded late in life delay the time when the estimated breeding values (EBV) are accurate enough. One way to increase the accuracy of EBV for these traits is to develop a multiple trait evaluation, which consists in taking advantage of the information from traits that can be recorded early in life and are correlated to the trait of interest. The information brought by the correlated traits enhances the accuracy of the evaluation of all the traits of interest. However, in some cases, simple multiple trait evaluations are not possible. Indeed, different correlated traits can be described by very different models: for instance, it is often important to consider the known sources of the heterogeneity of genetic and/or residual variances for some traits—e.g. for production traits [1, 2] or type traits [3]—or a permanent environmental effect for some other traits (e.g. mastitis occurrence or somatic cells count), or even a maternal effect. Software that deal with all of these more complex models together in a unique multiple trait analysis do not exist or may lead to a large increase in overall computing time. To overcome this problem, so-called “combined” best linear unbiased prediction (BLUP) evaluations have been developed [4], which consist of two steps and can be considered as an approximate multiple trait BLUP. First, corrected phenotypes are calculated for the traits of interest and their correlated traits by running univariate evaluations. The main purpose of this first step is to accurately estimate non-genetic effects by using models that are specifically adapted to the characteristics of each trait. The estimates of these non-genetic effects are then subtracted from the phenotypes and these corrected phenotypes are adjusted to a homogenous residual variance. In a second step, a multiple trait evaluation of the corrected phenotypes is run assuming a same simple model for all the traits. This approach results in more accurate EBV and a higher genetic gain in total merit [5, 6], especially for low-heritability traits.

Functional longevity (FL) is an important trait, which is receiving more and more attention as a relevant breeding objective to increase both the productive life and welfare of dairy cows [7]. However, FL has a low heritability and is difficult to predict because information for this trait on cows that are still alive is not complete. Genetic evaluation models for FL are also complicated by the need to include time-dependent explanatory variables, in a strongly nonlinear sire or sire-grand-sire model that is characterized by a non-normal residual distribution. This is why “combined” BLUP evaluations have been routinely used in France to improve the accuracy of genetic evaluations for FL, by adding information on traits that are correlated to FL and are considered as its early predictors [4]. In the French approach, breeding values of bulls for FL were first estimated using a survival analysis model that considers the length of the productive life of dairy cows adjusted for milk production [8, 9]. A pseudo-longevity record and its associated weight are derived for each cow in such a way that a standard BLUP animal model evaluation of these (weighted) pseudo-records approximately leads to the same EBV for the bulls [10]. This transformation can be seen as the basis of the extension of an animal model for sire longevity evaluations. In parallel, univariate genetic evaluations are performed for each trait that is correlated to FL, and the phenotypes corrected for non-genetic effects are estimated for each cow. Then, the pseudo-records for FL and the corrected performances of the traits considered as predictors of FL are used as phenotypes in a multiple trait genetic evaluation. This multiple trait genetic evaluation was implemented in 2010 [11] and provided EBV for FL, both for males and females, which were included in their total merit indices.

The inclusion of genomic information in genetic evaluations has led to an increase in genetic trend because own animal performances were no longer required to obtain accurate genomic (G) EBV [12]. Genomic evaluations are based on reference populations that consist of genotyped animals with own performance(s) or performance(s) of their relatives. As a result, genotyped cows can be selected or culled earlier based on their GEBV, often much before their own phenotype is collected. However, when early genomic selection is not accounted for, individual EBV as well as the genetic trends have been shown to be biased [13]. There is a strong competition in dairy cattle breeding programs to have accurate and unbiased GEBV of young males and females in order to reduce generation intervals and to obtain balanced genetic trends.

In recent years, single-step (SS) approaches, which consider simultaneously all the information available for all animals (pedigree, phenotypes, genotypes), have been developed to estimate GEBV. They reduce the bias in genetic trends and thus in GEBV and they increase the accuracy of GEBV by including information from ungenotyped animals [14–17]. However, computing requirements for large (e.g., national) applications may be quite substantial because of the slow convergence with some models, e.g., complex multiple trait models. As a consequence, only univariate SS evaluations (or multiple trait SS evaluations with traits described by a same model) are often implemented in practice, because the level of complexity and the computing time required when considering simultaneously several correlated traits described by different models are too great [17].

The aim of this study was to illustrate the benefits of a “combined” single-step (CSS) evaluation that can aggregate information from correlated traits [4–6]. These traits may be available for only a subset of the animals or described by different and more complicated models than a basic linear animal model (e.g., including heterogeneous residual variances). The benefits from the inclusion of correlated traits are essentially due to an increased accuracy without a major increase in computing time and even, in some cases, with a decrease in computing time [5, 6]. This study also investigates whether the prediction accuracy of a CSS evaluation is increased compared to that of a univariate SS (USS) evaluation. To illustrate the approach, we consider a joint evaluation combining six very different correlated traits in the Montbéliarde breed: FL, somatic cell score in milk (SCS), conception rate for heifers (CRH) and for lactating cows (CRC), clinical mastitis (CM) and udder depth (UD). A special attention is given to the accuracy of the evaluation of FL because improving FL meets both farmers’ and societal expectations ([18–20], and more generally those regarding animal welfare [21]).

## Methods

### Choice of traits

Functional longevity is difficult to predict with high accuracy because the true phenotype (length of productive life) is known only when the animal is dead. Many studies have shown that other routinely recorded traits are significantly correlated with FL, e.g. udder type traits [22, 23], fertility traits [4, 7], somatic cell score [4] and mastitis incidence [24]. These different traits are also correlated to each other and they can be considered as early predictors of FL [25–27]. Unlike the true FL, they are known from the first lactation (and even earlier in the case of heifer fertility), and thus they are good candidates to be included in a multiple trait evaluation to improve the accuracy of GEBV for FL, especially for animals that are still alive. In this study, we considered FL and five traits that are correlated to FL: somatic cell score (SCS), clinical mastitis (CM), conception rate for heifers (CRH) and cows (CRC) and udder depth (UD).

### Data

Records for the six traits mentioned above and pedigree information for the Montbéliarde cows used in this study were obtained from the French bovine national database [11, 28]. The dataset included all the cows born in 1998 and after, with a first phenotypic record for any of the six traits between 2000 and July 2021. In total, 2,837,644 cows had at least one record for one of the six traits studied. On average, a cow had performances for 3.7 traits. The pedigree file included 3,562,155 animals (with 3,520,038 females) among which 239,935 animals (3983 sires and 235,952 dams) had been genotyped with the BovineSNP50 BeadChip (Illumina Inc., San Diego, CA), and 15 groups of unknown parents.

Udder depth scores, ranging from 1 (very deep udder) to 9 (shallow udder), were collected by Montbéliarde breed technicians. Conception rate for heifers and CRC were based on the dates of artificial insemination (AI) and of the next calving of each cow. These are repeated traits representing the success (1) or failure (0) at each AI. Somatic cell score and CM were collected during monthly milk recording. Somatic cell score was expressed as the logarithm of the number of somatic cells per mL of milk as measured at each milk sampling test. Clinical mastitis indicated the presence/absence (1/0) of at least one mastitis event of a cow during lactation. Pseudo-records for FL were obtained after a routine genetic evaluation in France [11], which uses a Weibull model. The Weibull distribution [8, 9, 29] is a popular generalization of the exponential distribution which describes the hazard function with only two parameters (i.e., in our context, the probability for an animal to be culled at time t among those that are still alive at t − 1). In practice, standardized lactation productions are computed for each animal and compared to the average herd production, separately for first and later lactations. Then, the hazard function is corrected within herd and year for this average level of production. The *cumulative* hazard function during the productive life of the cow represents an aggregate measure of its risk of being culled, which is considered as a proxy of FL [10]. The genetic evaluation of FL is based on a sire-maternal grand sire model which allows to define FL “pseudo records” for cows to be included in the SS evaluation.

To contrast the accuracy of univariate and combined SS evaluations, an evaluation at the beginning of 2016 (without records for animals born in 2014 and 2015) was compared to the evaluation with all the data available in 2021. The elementary statistics of records for each trait are in Table 1.

**Table 1:** Distribution of performances by trait in 2016 and in 2021

Traits	2016			
	Number of performances	Number of animals	Mean number of performances per animal	Mean number of performances of dams of animals without performances in 2016
FL	1,409,428	1,409,428	1.00	1.55
CRH	2,232,913	1,506,695	1.48	2.33
CRC	4,016,853	1,267,628	3.17	4.73
SCS	3,550,513	1,637,909	2.17	3.59
CM	1,355,848	772,740	1.75	2.53
UD	918,654	918,654	1.00	1.45

Traits	2021				
	Number of performances	Number of animals	Mean number of performances by animal	Mean number of performances of animals without performances in 2016	Mean number of performances of dams of animals without performances in 2016
FL	1,963,384	1,963,384	1.00	1.00	1.58
CRH	3,027,361	2,050,240	1.48	1.45	2.36
CRC	5,472,488	1,746,630	3.13	2.52	4.74
SCS	5,101,670	2,316,890	2.20	1.94	3.69
CM	2,036,972	1,129,766	1.80	1.65	2.66
UD	1,319,119	1,319,119	1.00	1.00	1.49

*FL* functional longevity, *CRH* conception rate for heifer, *CRC* conception rate for cow, *SCS* somatic cell score, *CM* clinical mastitis, *UD* udder depth

The performances in Table 1 were used in the first step of a combined SS evaluation which consisted in six univariate SS evaluations, one for each trait. For each evaluation, records corrected for all non-genetic effects were obtained. For the traits with permanent environmental effects, pre-corrected records were grouped by animal and a weight based on the number of performances and the size of the contemporary group for each performance was associated to each corrected record. These records are called “pseudo-records”. For instance, for an animal $i$ with $x$ records for a same trait, the following formula was applied (here for ${Pseudo-record}_{i}$):

1 $${Pseudo-record}_{i}=\frac{recor{d}_{1}* c{g}_{{y}_{siz{e}_{1}}}+ \dots +recor{d}_{x}*c{g}_{{y}_{siz{e}_{x}}}}{ c{g}_{{y}_{siz{e}_{1}}}+ \dots +c{g}_{{y}_{siz{e}_{x}}}},$$

where $c{g}_{{y}_{siz{e}_{z}}}$ is the size of the contemporary group $z$.

### Estimation of the genetic correlations between traits by incorporating genomic information

Recent studies have included genomic information to re-estimate the genetic parameters and in particular the genetic correlations between traits, and it has been recommended to use all available genomic information for the genomic evaluations [30, 31]. For this reason, genomic correlations and heritabilities of the six investigated traits were computed by incorporating the genomic information obtained from the BovineSNP50 BeadChip (Illumina Inc., San Diego, CA, USA).

Covariance components were estimated using the following multiple trait sire model (with 6 traits):

2 $${\mathbf{y}}^{\boldsymbol{*}}=\mathbf{X}\mathbf{b}+\mathbf{Z}\mathbf{s}+{\mathbf{e}}^{\mathbf{*}},$$

where ${\mathbf{y}}^{\boldsymbol{*}}$ is the vector of pre-adjusted performances corrected for the dam’s EBV (derived from a standard univariate animal model), $\mathbf{b}$ is the vector of the fixed effect of birth year of the cow (in order to correct for genetic trends due to selection on correlated traits not accounted for in univariate evaluations), $\mathbf{s}$ is the vector of the sire effects, ${\mathbf{e}}^{\mathbf{*}}$ is the vector of random residuals, and $\mathbf{X}$ and $\mathbf{Z}$ are incidence matrices. To consider the different weights of pre-adjusted performances, a specific variance was attached to the residuals of each pre-adjusted record. The $\mathbf{G}$ matrix was constructed based on VanRaden’s first method [32]. Allele counts were centered by two times the observed frequencies and a small constant value of 0.01 was added to the diagonal elements to ensure a positive definite matrix. Variance and covariance components were estimated using the WOMBAT software [33].

Genetic correlations were calculated as:

3 $${r}_{g \mathrm{1,2}}=\frac{ 4{\sigma }_{s1,s2}}{\sqrt{{4\sigma }_{s1}^{2} * 4{\sigma }_{s2}^{2}}},$$

where ${r}_{g \mathrm{1,2}}$ is the genetic correlation between trait 1 and trait 2, $4{\sigma }_{s1,s2}$ is the genetic covariance between trait 1 and trait 2, ${4\sigma }_{s1}^{2}$ is the genetic variance of trait 1 and $4{\sigma }_{s2}^{2}$ is the genetic variance of trait 2.

The heritability of each trait was computed as the ratio between the genetic and phenotypic variance. Genomic correlations and heritabilities obtained for the six considered traits are in Table 2. Residual variances were inferred considering these heritabilities and genetic variances.

**Table 2:** Heritabilities (italics on the diagonal) and genomic correlations (off diagonal) between the traits considered

	FL	CRH	CRC	SCS	CM	UD
FL	*0.034*					
CRH	− 0.120	*0.022*				
CRC	− 0.446	0.548	*0.021*			
SCS	0.605	− 0.048	− 0.216	*0.393*		
CM	0.629	− 0.013	− 0.225	0.761	*0.022*	
UD	0.598	0.098	− 0.170	0.319	0.437	*0.630*

*FL* functional longevity, *CRH* conception rate for heifer, *CRC* conception rate for cow, *SCS* somatic cell score, *CM* clinical mastitis, *UD* udder depth

As expected, some correlations between traits are relatively high (absolute value > 0.4). In Table 2, FL is defined as the risk for a cow to be culled. This explains that the traits which are linked to diseases (UD, CM, SCS) are positively correlated with FL and fertility traits are negatively correlated with FL.

### Genomic evaluation models used with the hybrid single-step genomic BLUP software

The hybrid single-step (HSS)GBLUP software [34] is based on the model of [35] and has been used to compute the GEBV for all traits of animals from all French breeds.

The model used to describe the phenotypes for an ungenotyped animal is:

4 $${\mathrm{y}}_{\mathrm{ij}}={\mathrm{f}}_{\mathrm{p}}+{\sum }_{\mathrm{k}=1}^{nG}(({\mathrm{C}}_{\mathrm{ik}}-{\mathrm{J}}_{\mathrm{ik}})*{\mathrm{GG}}_{\mathrm{kj}})+ {\mathrm{a}}_{\mathrm{ij}} +{\mathrm{e}}_{\mathrm{ij}}.$$

For a genotyped animal, the model is:

5 $${\mathrm{y}}_{\mathrm{ij}}={\mathrm{f}}_{\mathrm{p}}+{\sum }_{l=1}^{nSNP}\left({\mathrm{g}}_{\mathrm{il}}*{\propto }_{\mathrm{lj}}\right)+{\mathrm{e}}_{\mathrm{ij}},$$

where ${\mathrm{y}}_{\mathrm{ij}}$ is the phenotype of animal $\mathrm{i}$ for trait $\mathrm{j}$, ${\mathrm{f}}_{\mathrm{p}}$ is the effect of the $\mathrm{p}$th level of the environmental effect where the $\mathrm{i}$th animal expresses its phenotype, ${\mathrm{GG}}_{\mathrm{kj}}$ is the effect of the $\mathrm{k}$th genetic group for trait j, $nG$ is the number of genetic groups (15 in this study), ${\mathrm{C}}_{\mathrm{ik}}$ is the raw contribution of the $\mathrm{k}$th genetic group to the $\mathrm{i}$th animal (${\sum }_{k=1}^{nG} {\mathrm{C}}_{\mathrm{ik}}=1$), ${\mathrm{J}}_{\mathrm{ik}}$ is the entry for animal $\mathrm{i}$ in vector $${\mathbf{J}}_{\mathbf{k}}=\left[\begin{array}{c}{\mathbf{J}}_{\mathbf{k}\_\mathbf{u}}\\ {\mathbf{J}}_{\mathbf{k}\_\mathbf{g}}\end{array}\right]$$, which contains the adjustment terms for the contribution of the $\mathrm{k}$th genetic group to animals (where ${\mathbf{J}}_{\mathbf{k}\_\mathbf{u}}={\mathbf{A}}_{\mathbf{n}\mathbf{g}}{{\mathbf{A}}_{\mathbf{g}\mathbf{g}}^{\mathbf{-1}}\mathbf{C}}_{\mathbf{k}\_\mathbf{g}}={-({\mathbf{A}}^{\mathbf{n}\mathbf{n}})}^{\mathbf{-1}}{\mathbf{A}}^{\mathbf{n}\mathbf{g}}{\mathbf{C}}_{\mathbf{k}\_\mathbf{g}}$ for ungenotyped animals, ${\mathbf{C}}_{\mathbf{k}\_\mathbf{g}}$ is the vector of contributions to the genetic groups of genotyped animals, and ${\mathbf{J}}_{\mathbf{k}\_\mathbf{g}}$ = ${\mathbf{C}}_{\mathbf{k}\_\mathbf{g}}$ for genotyped animals), ${\mathrm{a}}_{\mathrm{ij}}$ is the additive genetic effect of ungenotyped animal $\mathrm{i}$ for trait j, $nSNP$ is the number of single nucleotide polymorphisms (SNPs), ${\mathrm{g}}_{\mathrm{il}}$ is the centered genotype of individual $\mathrm{i}$ at the $\mathrm{l}$ th SNP, ${\propto }_{\mathrm{lj}}$ is the effect of allele 2 of the $\mathrm{l}$th SNP for trait j, ${\mathrm{e}}_{\mathrm{ij}}$ is the residual of the model.

At convergence, the GEBV were calculated as follows:

6 $${\mathrm{GEBV}}_{\mathrm{ij}}={\sum }_{k=1}^{nG}(({\mathrm{C}}_{\mathrm{ik}}-{\mathrm{J}}_{\mathrm{ik}})*\widehat{{\mathrm{GG}}_{\mathrm{kj}}})+\widehat{{\mathrm{a}}_{\mathrm{ij}}},$$

for the ungenotyped animals, where ${\mathrm{C}}_{\mathrm{ik}},$ ${\mathrm{J}}_{\mathrm{ik}}$ and ${\mathrm{GG}}_{\mathrm{kj}}$ are the same as (4) above, and

7 $${\mathrm{GEBV}}_{\mathrm{ij}}={\sum }_{l=1}^{nSNP}\left({\mathrm{g}}_{\mathrm{il}}*\widehat{{\propto }_{\mathrm{lj}}}\right),$$

for the genotyped animals.

### Combined single-step evaluation

The underlying principle of a “combined” genetic evaluation [4] was adapted to the SS evaluation. In a first step, univariate SS evaluations were run—each one with its specific model—to calculate the pseudo-records for each of the six traits considered in the study and their corresponding weights. Indeed, the traits considered here are described by different models. For instance, UD had three fixed effects (visit, age, and stage of lactation at the visit of the technician), three effects related to three sources of heterogeneity of variances (age at lactation stage, technicians year, visit) and the additive animal and residual effects, while CRH had seven fixed effects and no heterogeneity of variances (for more details on the different univariate models, see [36] but note that, in their study, the CRC, CRH, FL and CM traits used here are named CC1, HCO, LGF and MACL, respectively).

After the univariate evaluations step, for repeated traits, pseudo-records were computed as described above. Pseudo-records for each trait were used in the second step of the CSS evaluation with the following multiple trait model for all animals:

8 $${\mathrm{y}}_{\mathrm{ij}}^{*}=\mathrm{ye}+{\mathrm{a}}_{\mathrm{ij}}^{*}+{\sum }_{k=1}^{nG}\left(\left({\mathrm{C}}_{\mathrm{ik}}-{\mathrm{J}}_{\mathrm{ik}}\right)*{\mathrm{GG}}_{\mathrm{kj}}\right)+{\mathrm{e}}_{\mathrm{ij}}^{*},$$

for ungenotyped animals.

where ${\mathrm{y}}_{\mathrm{ij}}^{*}$ is the pseudo-record of animal $\mathrm{i}$ for trait $\mathrm{j}$, $\mathrm{ye}$ is a year effect (which in this case is equal to the effect of the birth year of animal $\mathrm{i}$), ${\mathrm{a}}_{\mathrm{ij}}^{*}$ is the additive genetic effect for trait $\mathrm{j}$ and ${\mathrm{C}}_{\mathrm{ik}}, {\mathrm{J}}_{\mathrm{ik}}$ and ${\mathrm{GG}}_{\mathrm{kj}}$ are as described above for Eq. (4) and ${\mathbf{e}}_{\mathbf{i}\mathbf{j}}^{\varvec{*}}$ is the residual effect and:

9 $${\mathrm{y}}_{\mathrm{ij}}^{*}=\mathrm{ye}+ {\sum }_{l=1}^{nSNP}\left({\mathrm{g}}_{\mathrm{il}}* {\propto }_{\mathrm{ij}}^{*}\right)+{\mathrm{e}}_{\mathrm{ij}}^{*},$$

for genotyped animals where ${\mathrm{g}}_{\mathrm{il}}$ is the genotype at SNP $\mathrm{l}$ and ${\propto }_{\mathrm{lj}}^{*}$ is the effect of SNP $\mathrm{l}$ on trait $\mathrm{j}$.

The objective of adding a year effect (ye) was to correct for potential selection bias that exists in the first step of the CSS evaluation where correlations between traits were ignored [6, 37, 38].

### Prediction assessment

With the objective to illustrate the benefits of a CSS evaluation, it was performed including all the information available for all the animals (columns with year 2021 in Table 1). The correlations between GEBV at the end of the univariate step and GEBV at the end of the multiple trait step were calculated for each trait.

In addition, another CSS evaluation was run *without* including any performance for FL in the dataset of year 2021. In such a scenario, GEBV for FL were predicted only from information coming from the other traits that are correlated to FL. To illustrate the impact of this source of information, correlations between GEBV for FL with the complete run and the run without records for FL—i.e., only relying on traits correlated to FL—were calculated.

Finally, in order to evaluate the quality of the genomic evaluations for FL on young animals, a USS evaluation and a CSS evaluation that mimic the situation at the beginning of 2016 were performed. For the animals born in 2014 and 2015, since they had no phenotype information, only genotype information was considered for the evaluation mimicked in 2016. The assumption was that these animals were genotyped soon after birth. For both SS evaluations in 2016, 118,714 genotyped animals were considered (Table 1).

Based on the information available at the beginning of 2016, GEBV of females born in 2014 and in 2015 were ranked from the best to the worst females and divided into deciles, separately for the two subpopulations (genotyped vs ungenotyped). For example, the first group of genotyped cows included the top 10% genotyped cows based on their GEBV for FL in 2016. Four separate cases were considered: GEBV from the USS and CSS evaluations in 2016 and for genotyped and ungenotyped heifers. These different cases were used to better visualize the quality of the different evaluations for FL, depending on the evaluation strategy used (with a univariate vs a combined single-step evaluation, for genotyped vs ungenotyped young females). For each of these four combinations, an average survival curve was calculated for each decile, using the Cox model of the Survival Kit software (v6.12) [39]. The Cox model is a non-parametric model that ensures a proper treatment of censored records (e.g., animals still alive or sold to another farm) without making any particular assumption about the shape of the survival curves. These survival curves for groups of cows born in 2014 or in 2015 were derived based on their status known in July 2021: they were either still alive—their length of productive life was treated as a censored record—or they were culled at a known date. To better quantify the difference in FL between groups, the observed mean differences between groups in number of productive days were calculated at different stages (see Additional file 1: Table S1). The reference group (for which the observed mean was globally set to 0) was the union of groups 5 and 6 of the ungenotyped heifers evaluated with the USS evaluation. All the other groups and types of population were compared to this reference group.

Moreover, in order to check the quality and impact of the within-herd standardization of FL for milk production with the current genetic evaluation using a Weibull sire model, the average milk records in first lactation after correction for lactation length were calculated for the cows grouped by GEBV deciles for FL separately for univariate and “combined” SS evaluations in 2016. By construction, it was expected that breeding values for FL were not influenced by the level of production.

In France, genomic evaluations have been used since 2009 in the three major dairy breeds (including the Montbéliarde breed) [40]. An intense selection on genomic breeding values has been applied on sires and bull dams, with a strong impact on the genetic trends for sires and candidate bull dams. This selection was based on a combination of the major economic traits [40, 41]. However, a large proportion of the young heifers has not been genotyped in most of the herds. Among the bulls born in 2011, the quality of the GEBV for FL of sires based on the CSS evaluation was compared to that based on the USS evaluation. For this comparison, four classes of bulls were created based on their GEBV obtained either from their USS or CSS evaluation in 2016. This year of evaluation was chosen because it corresponded to the year when daughters started to have records on at least one trait and because the aim was to assess the impact of the evaluation method (USS vs CSS) on the prediction of the bulls’ daughters. The best class was composed of sires with a GEBV for FL superior or equal to 1 genetic standard deviation ($1{\sigma }_{g}$) on the 2016 genetic base, while the next three sire groups included those with GEBV, respectively, between 0.5 and 1 ${\sigma }_{g}$, between 0 and 0.5 ${\sigma }_{g}$, and below 0 ${\sigma }_{g}$. The number of daughters per sire ranged from 101 to 4866 (with a mean of 737 ± 782 daughters per sire) for 114 sires. The length of the functional life of all the daughters (genotyped or not) which had a known or censored length of life was considered until July 2021. For each sire with at least 100 daughters, a survival curve based on their daughter’s information was constructed. Finally, an average survival curve of daughters was computed for each of the four sire groups. For this last step, the number of daughters of each sire was not considered to avoid an undue influence of the most popular sires. The comparison of the survival curves for the four groups of highly selected bulls should indicate the contribution of the correlated traits to identify the best bulls in terms of FL.

## Results

### Univariate vs multiple trait (= “combined”) evaluations

For each trait and for each of the three subpopulations (genotyped or ungenotyped cows, and genotyped sires), Table 3 shows the correlations between the GEBV obtained with the 2021 evaluations using the USS or the CSS approaches. For SCS, UD, CRH and CRC, in all three subpopulations, the correlations between GEBV are higher than 0.96. For FL and CM, these correlations depend on the subpopulation considered, i.e. for the ungenotyped females, the correlations are lower than 0.80 while for the genotyped animals (sires and cows), they are higher than 0.90. These results suggest that the inclusion of phenotypic information from the correlated traits had a much stronger impact on the two traits with the lowest heritability (FL and CM), especially for the ungenotyped females. This was expected and agreed with the theory of multiple trait linear models.

**Table 3:** Correlations between GEBV obtained from the USS and CSS evaluations in 2021 for each subpopulation and trait

Subpopulation	Traits					
	FL	CM	CRH	CRC	SCS	UD
Genotyped cows	0.91	0.91	0.98	0.98	1.00	1.00
Ungenotyped cows	0.77	0.71	0.98	0.96	0.98	0.98
Genotyped sires	0.94	0.93	0.98	0.99	1.00	1.00

*FL* functional longevity, *CRH* conception rate for heifer, *CRC* conception rate for cow, *SCS* somatic cell score, *CM* clinical mastitis, *UD* udder depth

**Fig. 1:**
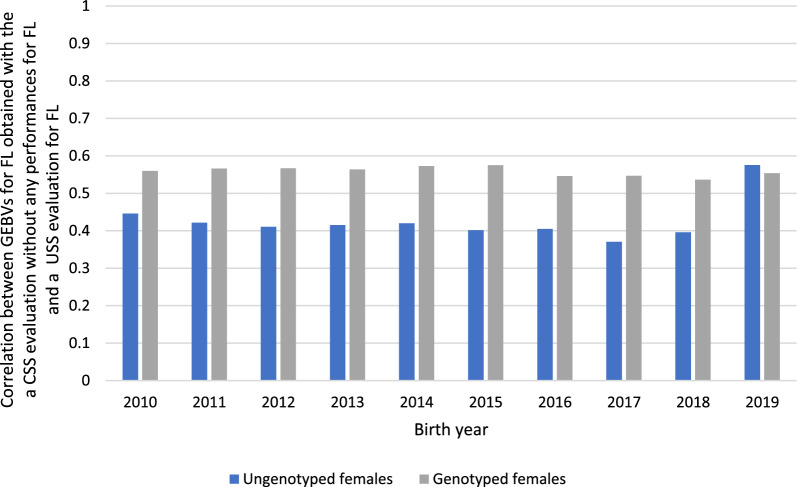
Correlations between GEBV for FL obtained with the CSS evaluation in 2021 without any performances for FL and those with a USS evaluation in 2021 for each population per year of birth

**Fig. 2:**
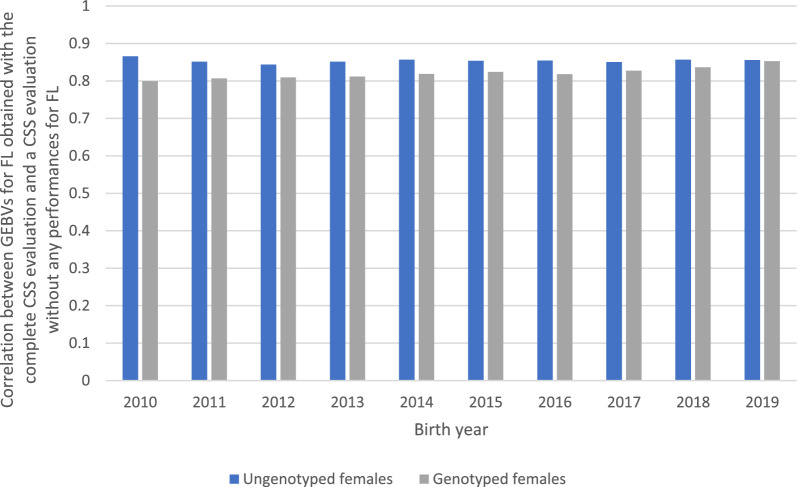
Correlations between GEBV for FL obtained with the complete CSS evaluation in 2021 and those with a CSS evaluation in 2021 without any performances for FL for each population per year of birth

Yearly correlations between the GEBV for FL obtained with the univariate evaluation and those obtained with a combined evaluation without performances for FL were also calculated for the females born between 2010 and 2019 (Fig. 1). For the genotyped females, the correlations ranged from 0.54 to 0.57 while for the ungenotyped females, they ranged from 0.37 to 0.45, except for the most recent year of birth (0.58 in 2019). These results show the substantial contribution of the traits that are correlated to FL to the estimation of the GEBV for FL with the CSS evaluation. The correlations between the GEBV obtained by including the information of all traits (including FL) in 2021 and those without performances for FL ranged from 0.80 to 0.87, with slightly higher values for the ungenotyped subpopulation (Fig. 2).

### Validation of early prediction of functional longevity

**Fig. 3:**
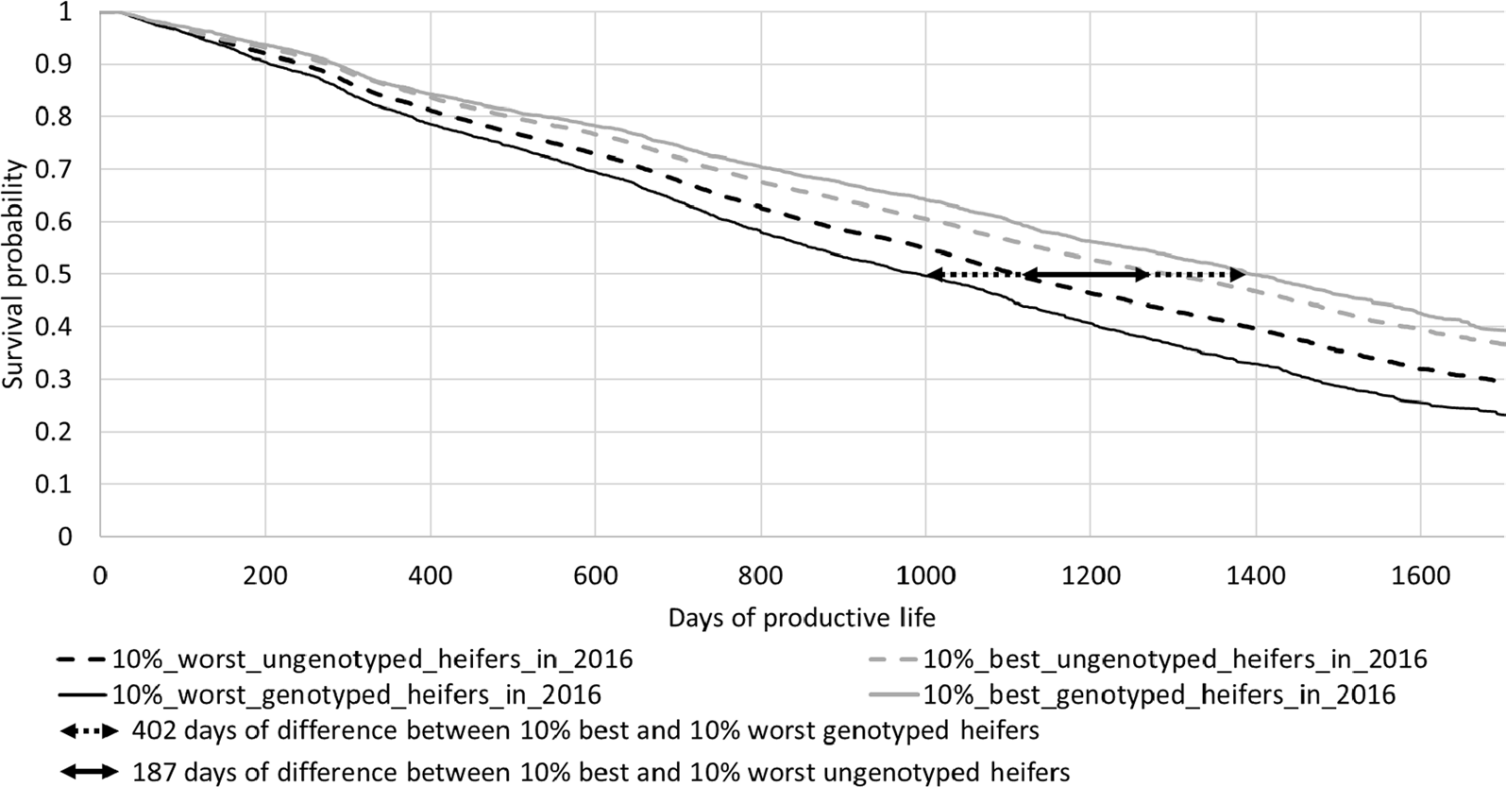
Survival curves of females born in 2014 and 2015 ranked according to their GEBV based on the USS evaluation in 2016

**Fig. 4:**
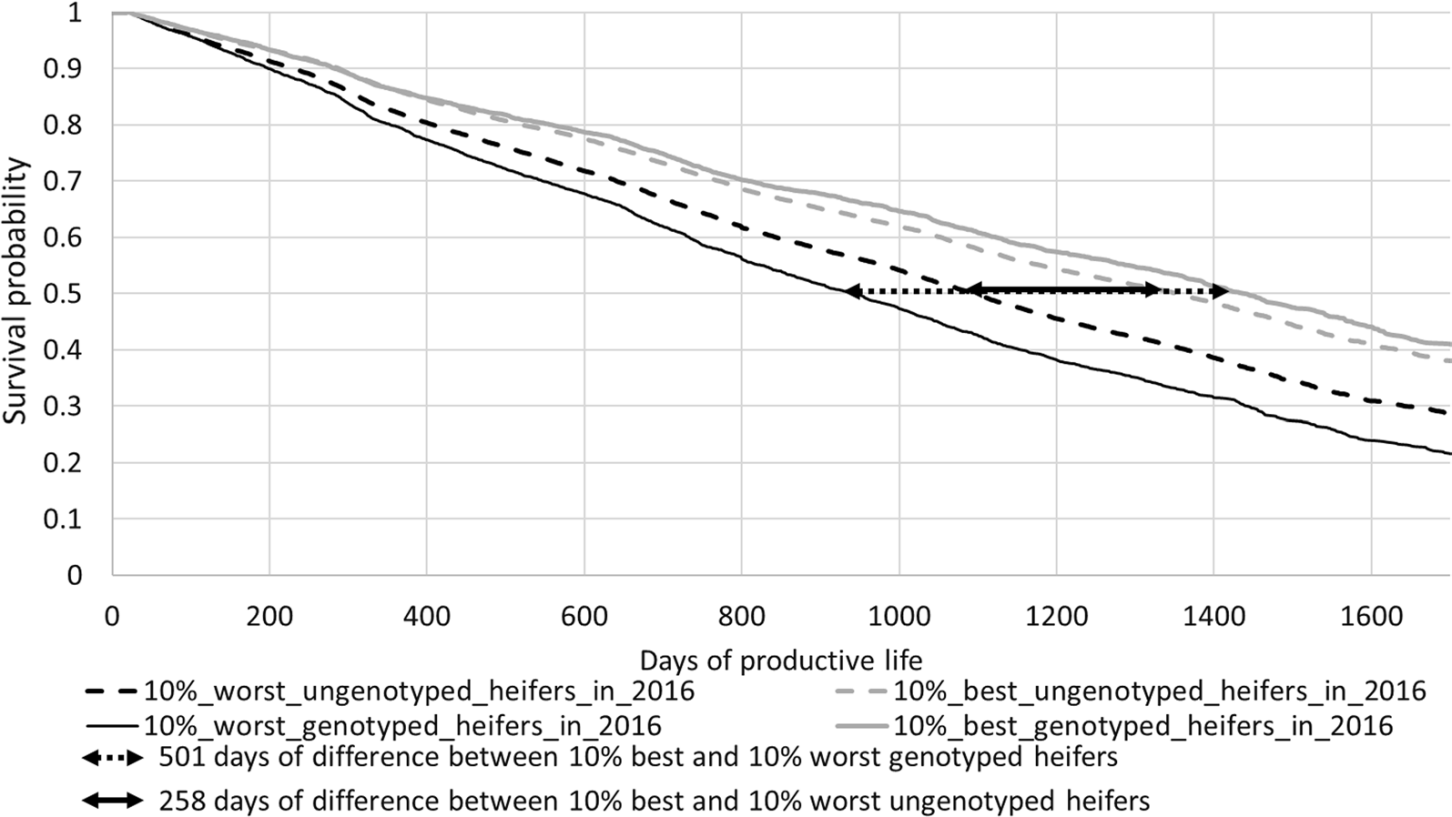
Survival curves of females born in 2014 and 2015 ranked according to their GEBV based on the CSS evaluation in 2016

Figures 3 and 4 show two graphs with survival curves calculated based on the GEBV for FL obtained with the USS evaluation (Fig. 3) or the CSS evaluation (Fig. 4) in 2016. For clarity, only the curves corresponding to the worst and best deciles are shown. These survival curves were calculated using all the information during the lifespan of these females, as known in July 2021 (but they are only shown up to a maximum of 1700 days because the end of the curves was not accurate). In fact, the 10 curves were distinct from each other, in the expected order (*not shown)* but the gap between curves was reduced for the best groups of females, and particularly for the best females that were evaluated with the CSS evaluation (curves in grey in Figs. 3 and 4). The two curves corresponding to the group of the best females are clearly distinct from each other: this reflects a difference between genotyped and ungenotyped females, regardless of the type of evaluation. This observation is also true for the group of the worst females. The gap in days between the USS and CSS curves increases with culling rate: for all groups and regardless of the genotyping status, the CSS evaluation leads to larger gaps between deciles than the USS evaluation. For instance, for the genotyped females, the 50% survival probability was reached 402 days earlier by the worst group than by the best group when these groups were defined using the USS evaluation, but it was reached 501 days earlier using the CSS evaluation.

**Fig. 5:**
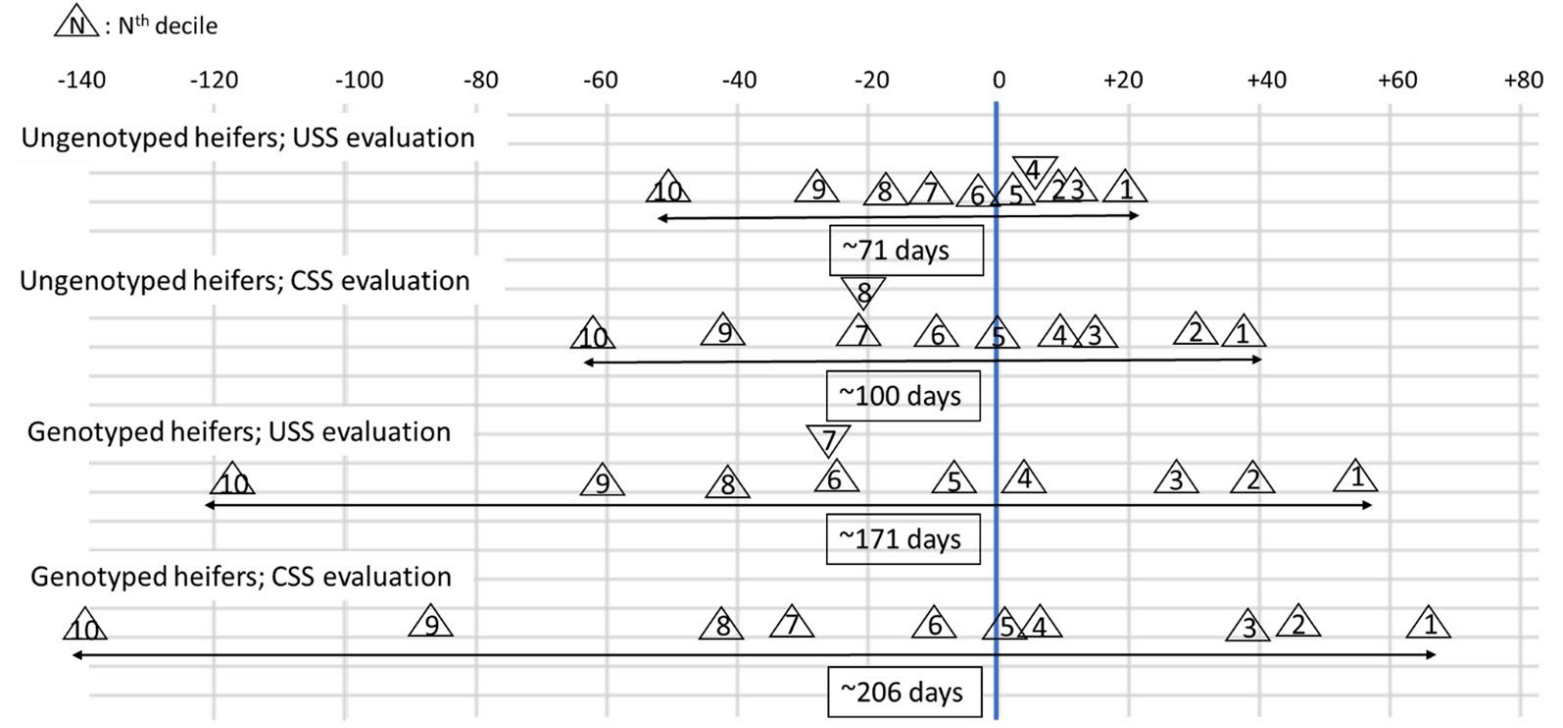
Observed mean difference in days of productive life between deciles of heifers based on USS or CSS evaluations

Figure 5 shows the observed mean difference up to 1600 days of productive life for heifers born in 2014 and in 2015 and ranked from the best (decile 1) to the worst GEBV (decile 10) obtained with the USS evaluation or CSS evaluation in 2016. Similar results for other periods of the productive life are shown in Additional file 1: Table S1. It was decided to show the results up to 1600 days of productive life because it corresponds to almost the maximum end of the productive life of Montbéliarde cows considered in the FL genetic evaluation in France. Figure 5 shows that the gaps between the worst deciles based on a univariate or “combined” analysis using genotypic information or not, are larger than for the best deciles. More generally, the difference between the average productive life of the best and the worst decile increased when using the CSS approach, regardless of the genotyping status of the female. The difference in average productive life between the best and the worst decile was even wider for the genotyped heifers (171 and 206 productive days with the USS and the CSS approach, respectively) than for the ungenotyped females (71 and 100 days). Furthermore, the difference in the gap between the extreme deciles appears to be almost the same (35 vs 29 days) between the USS and CSS evaluations for the genotyped and ungenotyped animals but, when expressed in %, the benefit from the CSS evaluation is more marked for the ungenotyped than for the genotyped subpopulation (29% vs 17%).

**Fig. 6:**
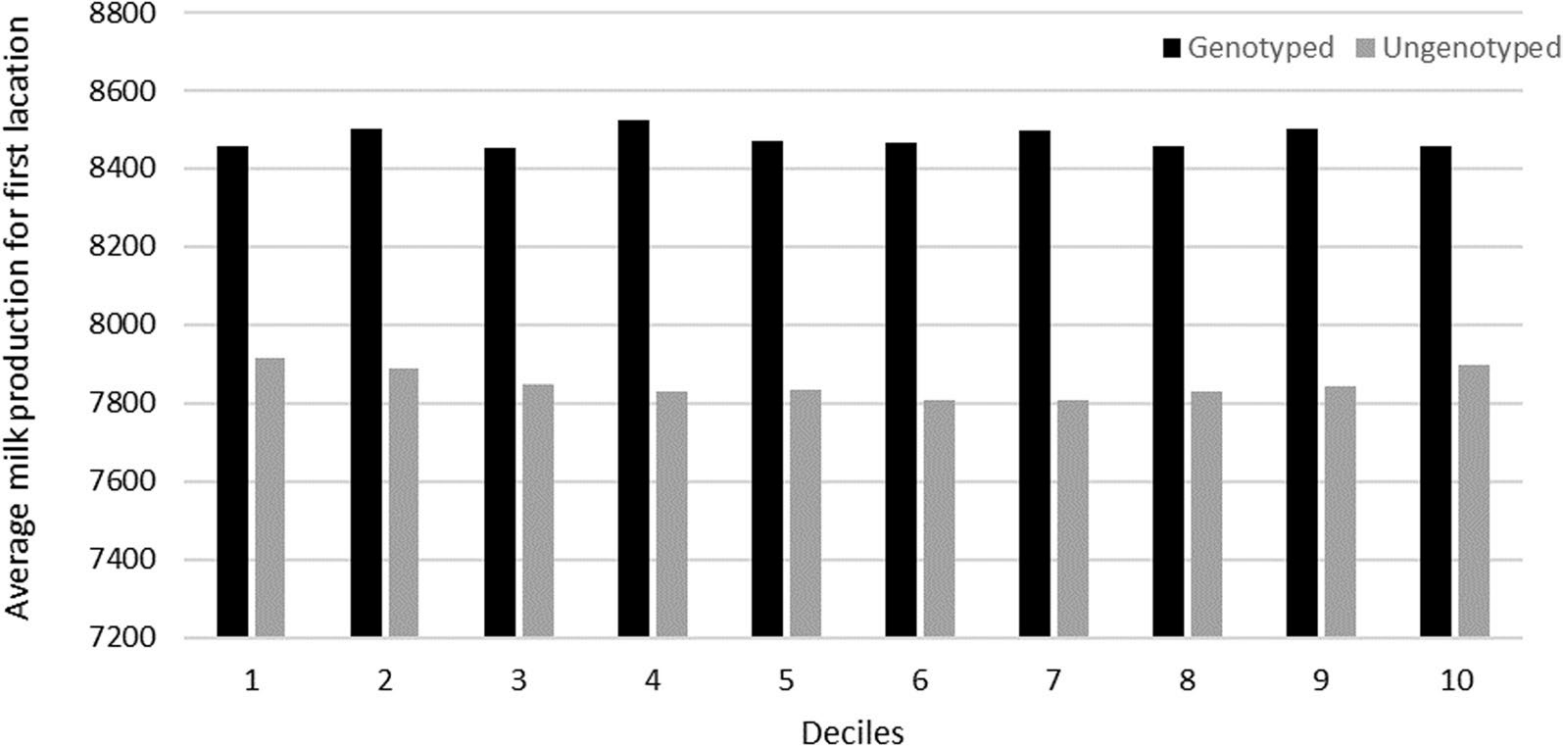
Average standardized milk production per GEBV deciles for functional longevity obtained with the USS evaluation in 2016 (decile 1 = the best animals for functional longevity)

**Fig. 7:**
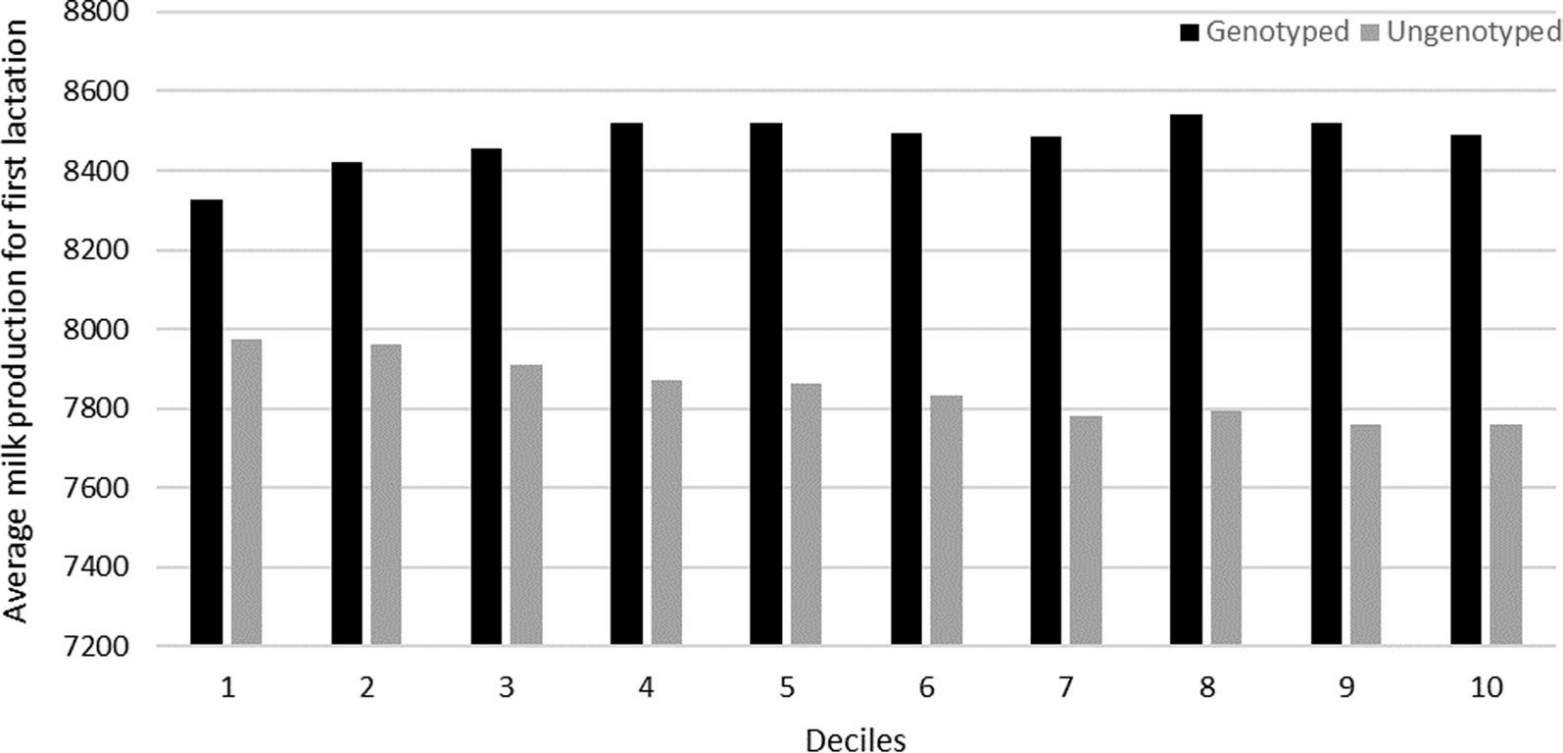
Average standardized milk production per GEBV deciles for functional longevity obtained with the CSS evaluation in 2016 (decile 1 = best animals for functional longevity)

Figures 6 and 7 compare the average standardized (305d) milk production in first lactation of genotyped and ungenotyped heifers according to their ranking (deciles) based on the results of the single-step evaluation for FL in 2016. Genotyped heifers have on average a higher milk production level in first lactation than the ungenotyped ones. This may be due either to the decision to genotype the daughters of the better cows, or to an early culling of the worst genotyped young females that are not kept in the herd as replacement heifers. When the classification is based on the results of the USS evaluation (Fig. 6), there is no apparent association between average production and class of functional longevity, as expected. In contrast, for the best deciles (1–3), the CSS evaluation reveals a slightly higher average milk production in first lactation for the ungenotyped cows and a slightly lower production for the genotyped ones (Fig. 7).

**Fig. 8:**
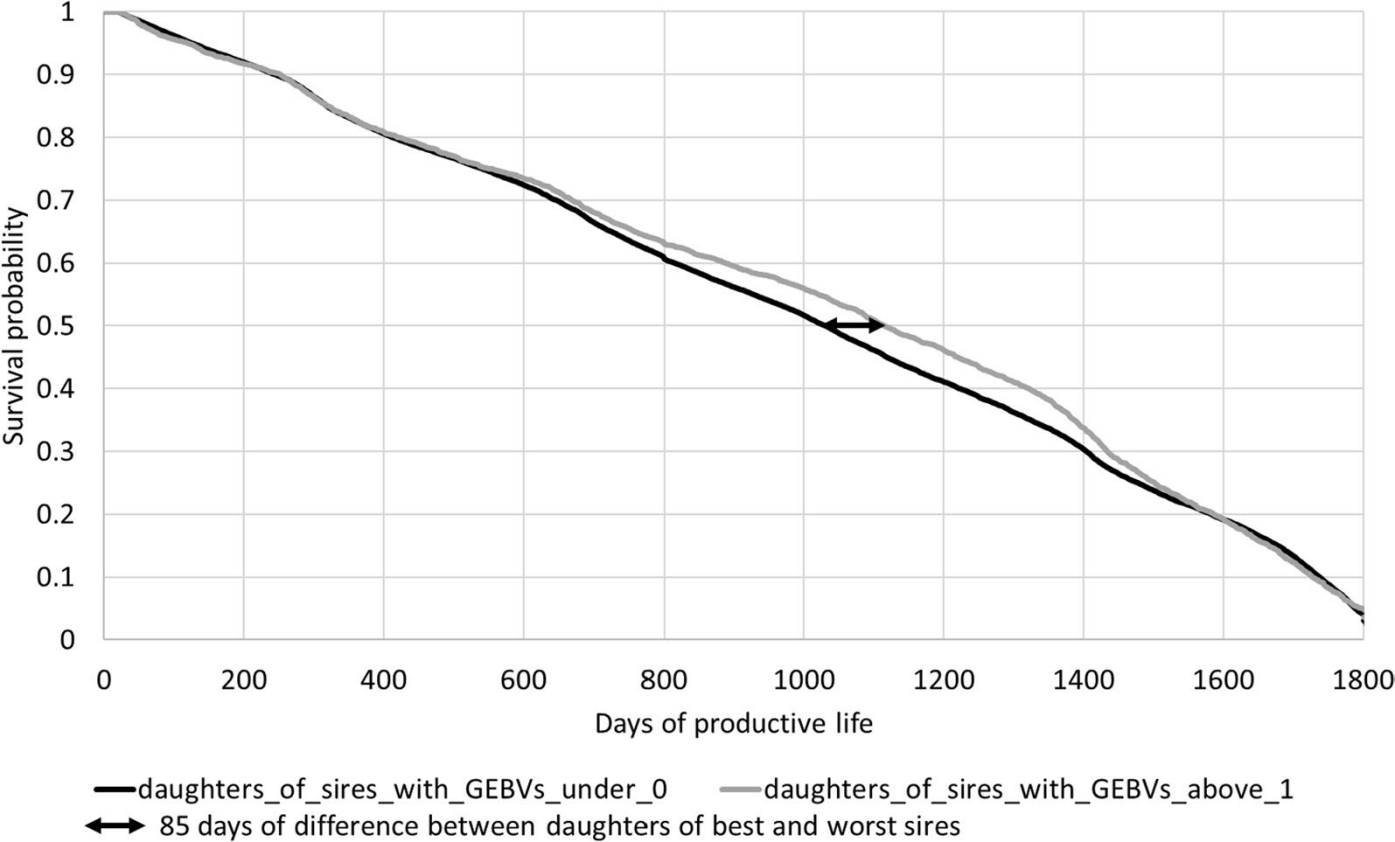
Survival curves of daughters of sires born in 2011 according to their GEBV based on the USS evaluation in 2016

**Fig. 9:**
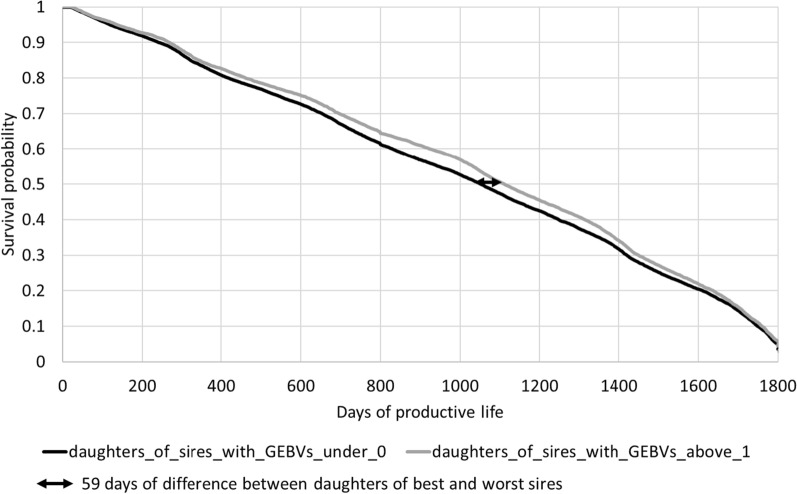
Survival curves of daughters of sires born in 2011 according to their GEBV based on the CSS evaluation in 2016

Figures 8 and 9 show two graphs with survival curves of daughters based on the rank of the GEBV of their sire, born in 2011. The class to which each sire belongs was calculated based on the USS evaluation (Fig. 8) or the CSS evaluation (Fig. 9) in 2016. As above, only the survival curves of the daughters from the best sires group and the worst sires group are shown. In both Fig. 8 and 9, a modest difference between curves can be observed. The gap between the USS and CSS curves increases with culling rate: at 50% survival, the difference in days of productive life between the two curves is 85 days for the USS evaluation but only 59 days for the CSS evaluation. However, within the CSS evaluation, the two curves separate from each other much earlier, possibly through the contribution of the correlated traits to the evaluation for FL.

## Discussion

Multiple trait evaluations have been developed to improve the accuracy of EBV in genetic evaluations through the inclusion of information on correlated traits, and to account for selection on these correlated traits [4, 5, 7]. Indeed, as long as the genetic parameters including correlations between traits are relatively well known, multiple trait evaluations are always more accurate than a linear combination of results from univariate analyses when records on animals are not available for all the traits and/or when the traits are described by different models [42].

Combined genetic evaluations have been proposed in order to alleviate the complexity of developing software that can handle joint evaluations of traits described by very different models. For example, Ducrocq et al. [4] developed a “combined” evaluation of dairy cows for production traits, somatic cell scores, female fertility, length of productive life and six type traits which are not described by the same model. Lassen et al. [5, 6] simulated different dairy cattle breeding goals that include a large list of traits and showed that a “combined” approach leads to higher genetic trends than univariate evaluations. Besbes et al. [43] proposed a “combined” evaluation of linear and nonlinear traits in laying hens that deals with a variety of traits (precocity, persistency, feed conversion ratio, longevity and a feather score).

With the development of genomic evaluation, “combined” *genomic* evaluations were not yet considered, probably because of the extra complexity involved. In addition, selection of young promising sires led to the emergence of biases in genetic evaluations [13]. The development of SS evaluations in the recent years decreased these biases due to pre-selection because they rely on all sources of information (pedigree and genomic information) [15, 44, 45]. Software that are able to handle multiple trait SS evaluations exist but mainly for traits that are described by the same model, or by models differing only for environmental effects, and for traits assumed to be continuous and normally distributed under a homoskedasticity hypothesis. Similarly to multiple trait genetic evaluation, a multiple trait SS approach improves the accuracy of GEBV for the traits evaluated together [46, 47].

A main limitation of the univariate genetic evaluations for FL is that they are inaccurate until a significant proportion of the daughters of a bull has been culled. When such a proportion is reached, the decision to select the bull for AI had already been taken much before. The joint analysis of FL and its correlated traits strongly improved the accuracy of the genetic breeding values for FL. It also increased their stability over time. A similar improvement was expected with a CSS evaluation. This was envisioned as an extension to SS of the “combined” genetic evaluation in order to benefit from the inclusion of information from correlated traits. As a result, CSS breeding values differed substantially from the USS values for FL and CM (Table 3). This was also the case for GEBV obtained with information on predictor traits but with and without any performances on FL (Figs. 1, 2). Indeed, the latter results showed that traits that are genetically correlated with FL contribute to the genetic/genomic evaluation for FL, much earlier when the true FL is still unknown. These observations illustrate the benefit of including information from correlated traits.

The survival curves (Figs. 3, 4) and the observed mean difference in days of productive life between groups (Fig. 5) showed that the best groups, and even more the worst groups of females, were distinct from each other, regardless of the type of evaluations. This demonstrates that SS evaluations are more accurate to estimate GEBV for FL. Moreover, genomic information was very important to better discriminate the worst and best groups of animals from each other, regardless of the type of evaluation. Thus, the output of the CSS evaluation appeared to be a better predictor to identify the best and the worst groups of females, regardless of their genotyping status. With a CSS evaluation, it is possible to better identify at birth the less robust heifers and to cull them, which may lead to interesting economic returns.

Our results show that, on average, the genotyped first lactation cows produced more milk on average than the ungenotyped cows, regardless of the type of evaluation. There are at least two interesting interpretations of this observation: either farmers decide to genotype only the heifers that they consider better than average or the heifers with the best GEBV for production traits receive some kind of preferential treatment. Indeed, the female cohort considered in 2014 and in 2015 corresponds in France to the beginning of a genotyping service to the farmers, in parallel to the genotyping proposed by the AI cooperatives which started a few years earlier, focusing on heifers with high genetic merit. Nevertheless, we found that the average milk production in first lactation was about the same, regardless of the functional longevity decile considered and their genotyping status. This is consistent with the model used for the underlying survival analysis which disentangles functional longevity from milk production [9]. However, the CSS evaluation (Fig. 7) shows a slightly different picture. The best genotyped cows (deciles 1 and 2 for survival GEBV) had a lower average milk production (about 200 kg less) in first lactation than for the other deciles. In contrast, the same deciles for the ungenotyped cows indicate a higher average milk production (about 200 kg more) than the other deciles. In other words, it seems that the CSS evaluation accounts better for the fact that elite genotyped heifers with the highest production may have more fertility or udder health problems [19, 48, 49], which can lead to the decision to cull them earlier [24]. Conversely, ungenotyped cows with a good fertility or udder are less susceptible to be culled. Overall, the inclusion of information on these correlated traits through the pedigree or genotypes improves the accuracy of the evaluations.

Regarding the results on genotyped bulls born in 2011, the benefit of the CSS evaluation was more limited than for the heifers, which may be explained by the fact that the bulls with progeny have been highly selected on a combination of all the important economic traits. This is a clear difference with the cows on any regular farm.

The joint analysis of correlated traits has a drawback, i.e. in general, the computing time of a CSS evaluation considering n traits is significantly larger than that of n USS evaluations. Each evaluation was parallelized on five compute nodes (computer server SuperMicro SYS-6028R-TRT, Intel Xeon 16 core processors) and with a very strict convergence criterion (correlation between iteration n and iteration n − 100 above 0.99 for USS evaluation and 0.999 for CSS evaluation). The USS evaluations took about 2–7 h to reach this criterion, depending on the traits. The corresponding time for the CSS evaluation was about 15 h. CSS evaluations (as well as multiple trait SS evaluations) improve the accuracy of GEBV and particularly for FL. This study considered the Montbéliarde breed which is the second largest dairy breed in France [28]. The computing time necessary for CSS evaluations for the Holstein breed is likely to be higher than for the Montbéliarde breed since it includes about 4 times more cows in milk recording. This may require to find compromises between computing time and gain in prediction accuracy resulting from the number of correlated traits considered. Other strategies such as deleting old genotypes and/or phenotypes can also be considered [50, 51], or less stringent convergence criteria.

In spite of these limitations, it appears that the proposed CSS evaluation provides a clear improvement of the predictability of FL, for both genotyped and ungenotyped cows. This would benefit to both the breeding schemes, which could improve the selection of bull dams on this trait, and the farmers for the choice of their replacement heifers. A similar approach could be applied in other similar situations. For example, this is the case for carcass traits (for calves, young bulls or cows) in dual-purpose breeds that could be combined altogether and with some conformation traits, such as the height of sacrum or chest width of cows, for example [52].

One of the anonymous reviewers indicated that extra validation of level and dispersion biases would have been possible by considering the correlations between GEBV for FL obtained with and without performances as displayed in Figs. 1 and 2. From these same sets of GEBV, the dispersion bias (measured as a regression coefficient) and the level bias can be computed similarly to the linear regression (LR) statistics proposed by [53].

## Conclusions

The proposed strategy to implement a “combined” single-step evaluation for functional longevity and correlated predictor traits showed very promising results in the case of the Montbéliarde breed. In particular, we showed that more accurate (G)EBV of both genotyped and ungenotyped heifers facilitate the selection of more robust cows, with a longer predicted productive life. The impact of such an approach is less visible on the selection of bulls, because the selection intensity of males for functional traits is already strong. The combined single-step approach can be extended to any complex phenotype resulting from elementary traits that are described by models that are too different to be considered directly in a multiple trait analysis.

### Supplementary Information

**Additional file 1: Table S1.** Mean observed difference in days of productive life between the extreme groups of heifers (worst and best deciles) at different stages of productive life. The data presented were derived from the survival curves obtained using a Cox model. The mean differences in days between extreme groups of heifers were calculated for the USS and CSS evaluations. The results are separated according to the genotyping status of heifers.

## Data Availability

Phenotypes, genotypes and pedigree data originated from the French National Animal Breeding database and they are owned by the French farmers. For this reason restrictions are applied to their availability.
