# Supplementary material for: Combined single-step evaluation of functional longevity of dairy cows including correlated traits
Source: Genet Sel Evol. 2023 Oct 25;55:75. doi: 10.1186/s12711-023-00839-6 (PMC10601146; doi:10.1186/s12711-023-00839-6)
Supplement: Supplementary file 1 — Additional file 1: Table S1. Mean observed difference in days of productive life between the extreme groups of heifers (worst and best deciles) at different stages of productive life. The data presented were derived from the survival curves obtained using a Cox model. The mean differences in days between extreme groups of heifers were calculated for the USS and CSS evaluations. The results are separated according to the genotyping status of heifers. [file 12711_2023_839_MOESM1_ESM.docx]

| **Days of productive life** | **Type of heifers** | **Difference between extreme groups with the USS evaluation** | **Difference between extreme groups with the CSS evaluation** | **Difference between the USS and CSS evaluations** |
| --- | --- | --- | --- | --- |
| 400 | Ungenotyped | 4.6 | 8.2 | 3.6 |
|  | Genotyped | 11.4 | 13.6 | 2.2 |
| 800 | Ungenotyped | 19.4 | 30.3 | 10.9 |
|  | Genotyped | 46.7 | 57.6 | 10.9 |
| 1100 | Ungenotyped | 36.1 | 53 | 16.9 |
|  | Genotyped | 88.7 | 107.4 | 18.7 |
| 1600 | Ungenotyped | 70.6 | 100.2 | 29.6 |
|  | Genotyped | 171.8 | 205.8 | 34 |
